# The Role of Protein Kinases in the Suppressive Phenotype of Myeloid-Derived Suppressor Cells

**DOI:** 10.3390/ijms26146936

**Published:** 2025-07-19

**Authors:** Aikyn Kali, Nurshat Abdolla, Yuliya V. Perfilyeva, Yekaterina O. Ostapchuk, Raikhan Tleulieva

**Affiliations:** 1 Laboratory of Molecular Immunology and Immunobiotechnology, M.A. Aitkhozhin Institute of Molecular Biology and Biochemistry, Almaty 050012, Kazakhstan; nurshata@gmail.com (N.A.); perfilyevayulya@gmail.com (Y.V.P.); katyostapchuk@gmail.com (Y.O.O.); raikhantleulieva@gmail.com (R.T.); 2 Almaty Branch of the National Center for Biotechnology, Almaty 050054, Kazakhstan

**Keywords:** myeloid-derived suppressor cells, protein kinases, immunosuppression, signaling pathways, context

## Abstract

Inflammation is a self-defense mechanism that controls the homeostasis of an organism, and its alteration by persistent noxious stimuli could lead to an imbalance in the regulation of inflammatory responses mediated by innate and adaptive immunity. During chronic inflammation, sustained exposure of myeloid cells to the various inflammatory signals derived from inflamed tissue could lead to the generation of myeloid cells with an immunosuppressive state, called myeloid-derived suppressor cells (MDSCs), which can exert protective or deleterious functions depending on the nature of signals and the specific inflammatory conditions created by different pathophysiological contexts. Initially identified in various tumor models and cancer patient samples, these cells have long been recognized as negative regulators of anti-tumor immunity. Consequently, researchers have focused on elucidating the molecular mechanisms underlying their potent immunosuppressive activity. As a key component of the signal transducing processes, protein kinases play a central role in regulating the signal transduction mechanisms of many cellular activities, including differentiation and immunosuppression. Over the past decade, at least a dozen kinases, including mechanistic target of rapamycin (mTOR), phosphoinositide 3-kinases (PI3Ks), TAM (Tyro3, Axl, Mer) family of receptor tyrosine kinases (TAM RTKs), mitogen-activated protein kinases (MAPKs), and others, have emerged as key contributors to the generation and differentiation of MDSCs. Here, we discuss the recent findings on these kinases that directly contribute to the immunosuppressive functions of MDSCs.

## 1. Introduction

Myeloid-derived suppressor cells (MDSCs) are heterogeneous populations of myeloid origin with potent immunosuppressive abilities, thereby disrupting T-cell proliferation and function. They have been identified to be a major obstacle in effective immunotherapy. Although inhibition of T-cell function by MDSCs has been studied widely, the spectrum of targeted cells is not limited to T cells, but can also be extended to B cells [1,2], NK cells, and DCs [3]. Moreover, the induction and recruitment of Tregs [4] and Bregs [5] and polarization of macrophages toward a tumor-promoting phenotype [6] can be attributed to the cellular mechanisms of immunosuppression by MDSCs. A growing body of evidence has demonstrated the non-immunological functions of MDSCs, including direct stimulation of tumor cell proliferation [7], enhancement of cancer stem cell properties [8], and promotion of pre-metastatic niche formation and metastasis [9]. Given their distribution across multiple organs and tissues, and the possible role of MDSC-derived exosomes in creating an immunosuppressive environment [10], MDSCs are now recognized as a critical component of the systemic or circulating tumor microenvironment (TME) [11].

Due to their shared phenotypic features with monocytes and neutrophils, the identity of MDSCs must be validated through functional tests. However, in general, in mice, MDSCs have been defined and enriched based on the combined expression of typical myeloid cell markers such as CD11b and Gr1, and to further distinguish the origin of the lineage, the Ly6C antigen is used to identify monocytic subpopulations (M-MDSCs) and Ly6G is for granulocytic subpopulations (G-MDSCs or PMN-MDSCs). In humans, however, characterization of MDSCs is more complicated than in their mouse counterparts, due to the absence of a direct equivalent to Gr1. Human MDSCs have been defined by excluding the maturity marker HLA-DR. Specifically, M-MDSCs are defined as CD11b^+^CD14^+^HLA-DR^−^/low CD15^−^, while G-MDSCs are identified as CD11b^+^CD14^−^HLA-DR^−^/low CD15^+^ or CD66b^+^. In addition, another subgroup termed early-stage MDSCs (e-MDSCs) has been described in humans and characterized as CD33^+^HLA-DR^−/low^CD11b^+^, and so far no mouse counterpart has been found [12].

The development and generation of MDSCs can be generally viewed as the chronic exposure of myeloid progenitor cells (which are at different stages of differentiation), and possibly committed myeloid cells, to the persistent signaling molecules released from a chronic inflammatory milieu, causing them deviate from the homeostatic differentiation program and resulting in the formation of activated neutrophils and monocytes with immunosuppressive abilities [13,14,15,16,17]. Although myeloid cells in this state of activation are beneficial in some autoimmune diseases [18,19], transplantation [20,21,22], and acute inflammation [23], they are destructive in the context of cancer and many other chronic inflammatory conditions, as these cells can be a major hurdle for the intrinsic immune system, which is committed to eradicating tumor cells, and also be a major challenge for effective immunotherapies [13,24,25,26].

**Figure 1:**
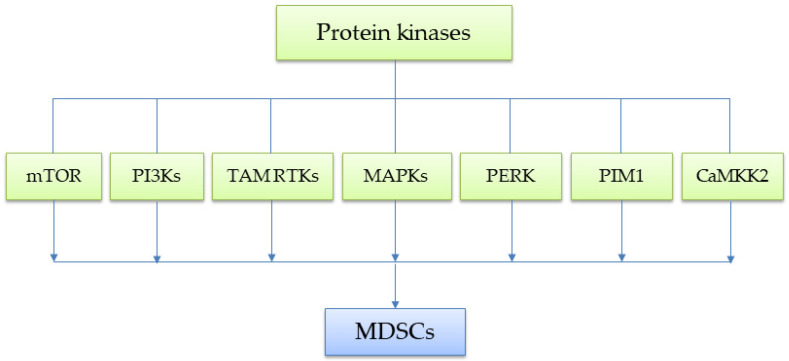
Schematic diagram illustrating the protein kinases that directly contribute to the immunosuppressive phenotype of MDSCs.

Protein kinases play a pivotal role in cellular signaling by catalyzing the phosphorylation of specific substrates, thereby regulating a wide range of cellular activities such as cell growth, metabolism, and immune responses. The human kinome consists of approximately 518 protein kinases, classified based on the amino acid residues they phosphorylate, reflecting their structural diversity and functional specificity [27]. Currently, protein kinases are broadly categorized into three major groups: (1) serine/threonine kinases (STKs); (2) tyrosine kinases (TKs); and (3) dual-specificity kinases (DSKs). Their dysregulation is implicated in numerous human diseases including cancer and various chronic inflammatory diseases, where targeted therapies have emerged as effective treatment strategies [28]. One of the major challenges in MDSC biology is understanding the kinase-mediated molecular mechanisms underlying their suppressive functions. A growing body of research has identified critical roles for at least a dozen signaling pathways regulated by protein kinases and their associated signaling axes). These include several STKs, such as mechanistic target of rapamycin (mTOR), mitogen-activated protein kinases (MAPKs), PKR-like endoplasmic reticulum (ER) kinase (PERK), Proviral integration site for Moloney murine leukemia virus-1 (PIM1), and Calcium/Calmodulin-dependent protein kinase kinase 2 (CaMKK2); phosphoinositide 3-kinases (PI3Ks), which are frequently activated by receptor tyrosine kinases (RTKs); and the TAM (Tyro3, Axl, and Mer) family of RTKs (Figure 1). In recent years, Bruton’s tyrosine kinase (BTK), a member of the TEC (tyrosine kinase expressed in hepatocellular carcinoma) family of non-receptor tyrosine kinases (NRTKs), has emerged as a key target in the suppressive phenotypes of MDSCs and TAMs. For an in-depth discussion of BTK, readers are referred to the excellent recent review [29] by Dr. Carson and colleagues, as it will not be covered in this review.

## 2. Protein Kinases in MDSCs

### 2.1. mTOR

Mechanistic target of rapamycin (mTOR) is a serine/threonine kinase that exists in two structurally and functionally distinct protein complexes, mTORC1 and mTORC2. By integrating environmental signals such as growth factors and nutrients, mTOR signaling regulates diverse biochemical and cellular functions, influencing processes such as metabolism, cell growth, immune responses, and autophagy, thereby orchestrating cellular homeostasis and responding to environmental changes. In addition, mTOR signaling has been shown to be involved in myeloid and lymphoid cell development, differentiation, and survival [30,31].

Under inflammatory and homeostatic conditions, mTORC1 plays essential roles by promoting glucose uptake and anabolic metabolism in M-CSF-mediated myelopoiesis [32]. Therefore, it can be assumed that mTOR-regulated glucose metabolism may play a role in the immunosuppressive activity of MDSCs. Indeed, mTOR has been shown as an intrinsic regulator of the differentiation and immunosuppressive function of M-MDSCs in alloskin-grafted and tumor-bearing mice models. Wu et al. [33] showed that rapamycin treatment or genetic deletion of mTORC1 but not mTORC2 resulted in a decreased number of M-MDSCs and immunosuppressive function. Unsurprisingly, through glucose metabolism, mTOR regulated these cellular activities, as evidenced by low glucose uptake, lactate production, and reduced expression of glycolysis-related enzymes by M-MDSCs when treated with rapamycin [33]. Another similar report [34] revealed that M-MDSCs, defined as CD45^+^CD11b^+^Ly6C^high^ and Ly6G^−^, from tumor tissues of mice showed overexpression of mTOR and increased glycolysis, as demonstrated by upregulated expression of glycolysis-associated genes and increased absorption of 2-(*N*-(7-Nitrobenz-2-oxa-1,3-diazol-4-yl)Amino)-2-Deoxyglucose (2NBDG), an indicator of glucose uptake. mTOR signaling was a key regulator of this increased glucose metabolism, as treatment of these tumor M-MDSCs with rapamycin resulted in a decrease in all of the above-mentioned glycolysis-associated features [34]. In the latter report, the effect of mTOR signaling is also probably mediated by mTORC1, as rapamycin preferentially targets the mTORC1 complex over mTORC2 [35].

Most of the mTOR studies use rapamycin, which preferentially inhibits mTORC1, to explore the role of mTOR in various cellular settings, including MDSC biology. However, as observed in a recent study [36], NK128, a new generation of mTOR inhibitors that target both mTOR complexes, played a more profound role in regulating M-MDSC differentiation compared to rapamycin [36]. An increased expansion and accumulation of M-MDSCs with active TLR7/IFN-α-mTOR signaling was found in a pristane-induced lupus mouse model, and it was associated with disease progression [36]. Metformin and mTOR inhibitors (rapamycin and NK128) were able to decrease the percentage of M-MDSCs while increasing the proportion of G-MDSCs. Adoptive transfer of metformin-treated M-MDSCs attenuated the disease symptoms. Among the three mTOR inhibitors, metformin and NK128 showed a more profound effect in regulating M-MDSC differentiation compared to rapamycin [36]. Although the authors did not perform the standard functional assay to assess the immunosuppressive ability of the MDSC populations found in this mouse model, they generated in vitro MDSCs in the presence of well-established MDSC-inducing cytokines, GM-CSF and IL-6 [37]. The molecular mechanism of this M-MDSC differentiation was dependent on interferon regulatory factor-8 (IRF-8), an important transcription factor involved in the differentiation of myeloid cells from hematopoietic stem cells [38]. Although elevated levels of IRF-8 have been shown to play a critical role in TLR7/IFN-α-mediated differentiation of M-MDSCs in a lupus model [36], earlier studies have identified IRF-8 as a negative regulator of MDSC accumulation and the development of suppressive phenotypes in various tumor models and cancer patients [39,40]. This apparent contradiction in IRF-8 function may, in part, be explained by differences in the underlying pathophysiological conditions and the predominance of specific MDSC subsets in each context. For example, in the lupus microenvironment, factors such as IFN-α and TLR-7 agonists could shift the MDSC differentiation towards M-MDSCs along with an increased level of IRF-8 while decreasing G-MDSCs [36]. In contrast, studies in tumor-bearing mice have shown a more pronounced loss of IRF-8 in G-MDSCs compared to M-MDSCs [39]. Of note, metformin is an agonist of AMP-activated protein kinase (AMPK), a crucial enzyme that regulates cellular energy metabolism and immune responses [41]. As an upstream molecule of mTOR, activation of AMPK can inhibit mTOR signaling. Moreover, some reports have demonstrated AMPK as a negative regulator of the immunosuppressive functions of MDSCs, as its activation inhibits most of the MDSC-associated transcription factors, including STAT3, C/EBPβ, and NF-κB [42]. In addition, metformin, through activation of AMPK, inhibits migration and accumulation of MDSCs [43]. However, several studies have reported contradictory results, showing that AMPK activation is required for the immunosuppressive functions of MDSCs [33,44,45].

On the other hand, in the context of some autoimmune diseases, mTOR signaling may play a less important role in terms of the suppressive function of MDSCs. For example, Chen et al. [46] reported that MDSCs from two different hepatic injury models (CIH and PIH) constitutively expressed mTORC1, as indicated by increased phosphorylation of S6. Surprisingly, these cells, from the liver and spleen, acquired an even greater suppressive phenotype, both in vivo and in vitro, when these cells were treated with rapamycin compared to MDSCs generated in these hepatic injury models, and these mTORC1-deficient MDSCs exerted protective effects against immune-mediated hepatic injury and showed improved survival [46]. In this study, it is not known which subpopulation of MDSCs was affected most by mTOR-mediated signaling, as the authors examined the total MDSCs without separating the subpopulations. Importantly, the active mTORC1 did not diminish the suppressive function of MDSCs in this hepatic injury model, but instead its absence potentiated the suppressive ability of MDSCs [46], indicating the complexity of the immunosuppressive program in this model system. Also, it remained unknown whether mTORC1 inhibition shifted MDSC differentiation towards G-MDSCs, as reported recently [47]. Similarly, rapamycin-treated MDSCs exhibited a protective effect in a mouse model of acute kidney injury (AKI) [47]. Although AKI is not a model for autoimmune disease, it involves significant immune activation, including infiltration of T cells, among other immune cells, into the injured kidney and subsequent exacerbation of tissue damage [48]. Zhang et al. demonstrated that adoptive transfer of rapamycin-treated MDSCs, generated in vitro by GM-CSF and IL-6, limited T-cell infiltration and significantly improved renal function. Interestingly, in vitro experiments further showed that mTOR inhibition regulated the induction of MDSCs towards the CD11b^+^Ly6G^+^Ly6C^low^ G-MDSC subset, enhancing their immunosuppressive activity through upregulation of Arginase-1 and iNOS [47]. Taken together, these studies suggest that active mTORC1 signaling may play a more significant role in generating M-MDSCs with potent suppressive activity compared to G-MDSCs. This difference may be partly attributed to mTORC1’s critical involvement in M-CSF-driven myelopoiesis, particularly within the monocytic lineage [32].

mTOR is a critical downstream effector within the highly conserved PI3K-Akt signaling network. Hyperactivation of the PI3K/Akt/mTOR pathway is frequently observed in various cancer types, where it contributes to tumor cell survival, proliferation, and growth [49]. Beyond its role in oncogenesis, this signaling cascade is also pivotal in regulating immune cell function. It influences both innate and adaptive immune responses by integrating diverse extracellular signals to control key cellular processes such as metabolism, proliferation, and differentiation. These regulatory mechanisms are essential for mounting effective and balanced immune responses [50,51,52]. The activation of the PI3K/Akt/mTOR pathway has been found to be associated with increased immunosuppressive functions of MDSCs in different tumor models [53,54,55]. Increased levels of G-CSF in serum from tumor-bearing mice activated the PI3K/Akt/mTOR signaling pathway and immunosuppressive functions of G-MDSCs generated in the context of a B16F10 melanoma tumor model. Rapamycin treatment decreased the percentage of G-MDSCs in peripheral blood and downregulated Arg1 expression both in in vivo and in vitro conditions [53]. Although the concentration of GM-CSF showed a gradually decreasing tendency in the serum of tumor-bearing mice with tumor progression in the melanoma model [53], treatment of monocytes with GM-CSF was required for the licensing of monocytes to be suppressive cells [54]. Moreover, working together with IFN-γ-mediated signaling, GM-CSF resulted in the generation of M-MDSCs with an even more suppressive phenotype characterized by the activated PI3K/Akt/mTOR signaling axis [54]. As an important molecule in regulating the process of emergency myelopoiesis during infection and inflammatory conditions, the role of GM-CSF in inducing MDSC differentiation and generation both in humans and mice has been well established [56,57,58]. It has been reported that varying quantities of GM-CSF can influence the differentiation and function of myeloid cells differently [59]. Even as low as 0.3 ng/mL of GM-CSF induced monocytes with suppressive ability [54]. Thus, it is not surprising that varying levels of GM-CSF in the TME, combined with multiple other factors (e.g., IL-6, IL-1β, VEGF, and TGF-β) could generate diverse MDSCs with differing suppressive abilities [60].

In general, mTORC1, sensitive to nutrients and energy, promotes growth and metabolism, whereas mTORC2, responsive to growth factors, regulates cell survival and cytoskeletal organization [30,31]. Studies have shown that mTORC2 is critically involved in the differentiation of myeloid cells. For example, Hallowell et al. reported that mTORC2 is important for M2 macrophage differentiation and function while not necessary for M1 differentiation [61]. Another similar study revealed that mTORC2 is required for TAMs to promote tumor growth [62]. The last two studies suggest a possible role for mTORC2 in the generation of MDSCs with a suppressive phenotype. Research has also suggested that mTORC2 may play a role in DC differentiation and its absence promotes the proinflammatory function of DCs. A recent study reported that intra-tumoral injection of mTORC2-deficient DCs results in delayed B16 melanoma growth and a reduced frequency of MDSCs in the TME [63]. However, the specific role of mTORC2 in the regulation of the suppressive phenotype of MDSCs remains largely unexplored.

### 2.2. PI3Ks

Unlike typical protein kinases that directly phosphorylate proteins on serine, threonine, or tyrosine residues, phosphoinositide 3-kinases (PI3Ks) produce second messengers (PIP_3_) that use their pleckstrin homology (PH) domains to attract downstream effectors such as Akt/PKB and mTOR and thereby transduce signals important in cell growth, survival, metabolism, and migration. Based on their structure and regulation, PI3Ks are broadly divided into three classes (I-III) and consist of eight isoforms. Class I PI3Ks are the most studied due to their significant involvement in cancer and immune regulation. For example p110δ and p110γ isoforms are highly enriched in leukocytes and play important roles in activating innate immune cells during inflammation and T- and B-cell development [64]. Elevated PI3K signaling activity has been found in multiple cancer types and is considered as a hallmark of cancer [65].

Several recent reports have convincingly demonstrated the crucial role of PI3K signaling in the suppressive phenotype of MDSCs. Non-selective pan-inhibitors of PI3Ks such as Wortmannin and LY294002 induce differentiation of M-MDSCs into the mature phenotype along with decreasing suppressive ability [66]. In a recent human study, PI3K/AKT signaling was found to be the master regulator of the suppressive function of monocyte-derived M-MDSCs generated with a low dose of GM-CSF and IL-6. The results from in vitro studies were supported by the phenotypic and functional resemblance between in vitro-generated and cancer patient-derived M-MDSCs. The mechanistic studies showed that inhibition of PI3K using chemical inhibitors, Wortmannin and LY294002, reversed the immature phenotype to a more mature phenotype, as evidenced by increased HLA-DR surface receptors. These phenotypic changes were further confirmed by the functional study where T-cell suppression was rescued when co-cultured with PI3K-inhibited M-MDSCs [66]. Another important finding of this study is that PI3K signaling induces an early and irreversible commitment to M-MDSC development. Inhibition of PI3K 4–24 h before cell harvesting did not increase HLA-DR expression, whereas inhibition 3–7 days prior was effective. This suggests that PI3K-driven signaling establishes a differentiation program that locks cells into a suppressive MDSC state, potentially limiting the effectiveness of late-stage therapeutic interventions.

Comparative analysis of molecular features between pathologically activated MDSCs and their normal counterparts provides a valuable tool for identifying critical molecular distinctions. Using systems biology analysis Gato-Cañas et al. [67] compared myeloid DCs and non-cancerous (NC-MDSCs) and cancerous MDSCs (C-MDSCs). As a result, they found a distinct kinase signature in these cells, including activation of the PI3K-AKT, SRC, ERK, and ERK pathways. Flow cytometry and immunoblotting analyses validated the proteomic findings, demonstrating increased expression of active AKT and phosphorylated ERK (pERK) proteins in C-MDSCs compared to DCs and NC-MDSCs. Furthermore, functional assays using specific siRNAs and small-molecule inhibitors—X for AKT and PD0325901 for MEK—revealed that the AKT and MEK-ERK pathways are critical for C-MDSC survival and differentiation, respectively [67].

Previous studies have shown that p110δ and p110γ, two isoforms that belong to class I PI3Ks, are abundantly expressed in hematopoietic cells including macrophages, neutrophils, and T cells, suggesting their critical roles in regulating myeloid cell activities. PI3Kγ is highly expressed in myeloid cells but not in tumor cells, making it an attractive research target in myeloid cells associated with immunosuppressive function. Indeed, it has been found that PI3Kγ plays a central role in controlling immune-stimulating and immune-suppressing states in macrophages [68]. The specific molecular mechanism revealed that PI3Kγ signaling through Akt and mTOR downregulates NFκB activation while stimulating C/EBPβ activation; the latter promotes an immunosuppressive transcriptional program during inflammation and tumor growth [68]. Among the various transcription factors associated with MDSCs, C/EBPβ has emerged as a key regulator of their potent suppressive functions [37,69], likely due to its role in driving *Arg1* and *IL-10* expression through direct binding to their promoter regions [70,71]. However, the kinase-dependent regulation of C/EBPβ in MDSC biology remains poorly understood. To date, only one recent study has demonstrated that *Arg1* and *IL-10* expression in MDSCs is controlled via the Fyn–STAT3–C/EBPβ signaling axis in a model of abnormal pregnancy with *Toxoplasma gondii* infection, as well as in human decidual MDSCs infected with *T. gondii* [71]. Fyn, an NRTK belonging to the Src family of kinases (SFKs), was shown to interact with Tim-3, leading to STAT3 phosphorylation and promoting the suppressive phenotype of MDSCs [71]. Given the central role of C/EBPβ in MDSC function, further studies are warranted to elucidate the kinase-dependent mechanisms governing its regulation. In the context of MDSCs, inhibition of PI3K δ/γ isoforms with IPI-145 led to reduced phosphorylation of AKT and S6 in G-MDSCs and thereby attenuated the suppressive activity of these cells mediated by Arg1 and iNOS [72]. Although preliminary research shows that non-specific pan-PI3K inhibitors effectively target the suppressive functions of myeloid-derived suppressor cells [66], this approach may not yield optimal outcomes in clinical settings, particularly due to its impact on normal T lymphocyte function [72]. Studies have shown that selectively targeting PI3K p110δ could efficiently inhibit the proliferation and survival of Tregs while not disturbing conventional T cells’ function [73]. Earlier, Ali et al. found that G-MDSCs derived from genetically p110δ-deleted mice lost their suppressive ability against anti-CD3-stimulated T cells compared to wild-type G-MDSCs [74]. However, blocking of PI3K p110δ using its selective inhibitor PI-3065 does not directly target the MDSC numbers nor affect the suppressive phenotype of MDSCs [75]. The discrepancy between these two studies can be partially explained by the reversibility of the inhibitor treatment, as suggested by the authors [75]. Nevertheless, it seems that selective inhibition of PI3Ks, especially p110δ in MDSCs, may promise a targeted approach to modulate the immunosuppressive TME, potentially enhancing anti-tumor immunity and improving the efficacy of cancer immunotherapies.

### 2.3. TAM Receptor Tyrosine Kinase

TAM receptor tyrosine kinases (TAM RTKs) are a subfamily of RTKs and consist of three members: TYRO3, AXL, and MERTK. They are activated by two main ligands: Growth Arrest-Specific 6 (GAS6) and Protein S (PROS1), which bind to phosphatidylserine (PtdSer) exposed on the surface of apoptotic cells or activated immune cells. Under physiological conditions, TAM RTKs are expressed in a wide range of cell types, including both hematopoietic cells and non-hematopoietic cells. Although their functions seem to be redundant and substituted with other RTKs during the early stage of development, during inflammation, upregulation of TAM RTKs by myeloid cells, including DCs and macrophages, plays a critical role as a negative regulator of inflammation involving the restoration of homeostasis by limiting excessive immune responses, promoting phagocytosis of apoptotic cells, and eventually recovering tissue function [76]. Among these three members, it has been shown that MERTK seems to play a particularly essential role in the resolution of inflammation by enhancing efferocytosis, switching the cytokine profile towards proresolving mediators, and suppressing neutrophil activation and neutrophil extracellular trap (NET) formation [77].

However, in the context of cancer, activated MERTK in macrophages polarizes them into a tumor-promoting phenotype and supports disease progression by creating an immunosuppressive microenvironment [78]. Unsurprisingly, the anti-inflammatory role of MERTK has garnered interest in MDSC biology. In a lung transplantation model, M-MDSCs with activated MERTK effectively facilitated the resolution of inflammation in ischemia/reperfusion injury by clearing the apoptotic neutrophils at injury sites, while M-MDSCs derived from MERTK^−/−^ mice failed to exhibit an immunosuppressive effect [79]. Recently, Holtzhausen et al. [80] demonstrated TAM RTKs as key contributors to the immunosuppressive activity of MDSCs in a murine melanoma model and in human melanoma patients. At first, the authors showed the overexpression of all three TAM RTKs and their ligands in tumor-bearing mice. Then, the spleen MDSCs from Mertk^−/−^, Axl^−/−^, and Tyro3^−/−^ mice showed decreased expressions of suppressive mediators such as arginase-1, iNOS TGF-β, IDO, and ROS compared to the MDSCs derived from wild-type mice, and the functional assay was consistent with these results. Mechanistic studies further revealed that this suppressive activity is mediated by STAT3 phosphorylated on the serine residue [80].

Although the family members of TAM RTKs are not considered as oncogenic, their expression in cancer cells endows them with a survival advantage under stressful conditions such as chemo-drug treatment. Considering the crucial role of MERTK and other members of TAM RTKs in creating an immunosuppressive microenvironment and enhancing the survival of cancer cells, it can be suggested that targeting these kinase members in a time-controlled and cancer-specific manner could be beneficial in improving cancer patients’ disease outcome while not disturbing the homeostatic balance of the immune system [81].

### 2.4. MAPKs

Mitogen-activated protein kinases (MAPKs) are a family of STKs that are activated by various extracellular stimuli, including cytokines, growth factors, and extracellular matrix molecules, and regulate a wide variety of cellular activities, such as cell proliferation, differentiation, survival, and apoptosis. In mammalian cells, the MAP kinases consist of four major downstream effector kinases: ERK1/2, JNK 1/2/3, p38 α/β/γ/δ, and ERK3/4/5. Their activation mode is dependent on the nature of external stimuli; for example, ERK pathways are activated by growth factors and mitogens, while JNK and p38 pathways are stimulated by various stress factors, including inflammatory cytokines, UV radiation, and high osmotic stress [82].

Aberrant expressions of these MAPKs are found in various pathological conditions such as cancer, neurodegenerative disorders, and inflammatory diseases including rheumatoid arthritis and inflammatory bowel disease [83]. Physiologically, the family members of MAPKs are essential in controlling the balance between expansion, survival, and differentiation of myeloid progenitor cells, indicating their potential implication in emergency myelopoiesis and subsequent expansion of myeloid cells with suppressive functions [84]. In an LL2 tumor model, RNA sequencing analysis revealed that increased activity of MAPK signaling was observed in PMN-MDSCs and M-MDSCs derived from tumor tissue compared to those derived from the spleen. In vitro experiments further showed that inhibition of ERK1/2 and JNK with SCH772984 and SP600125, respectively, significantly increased apoptosis of both MDSC populations, while inhibition of p38 with Ly2228820 did not cause cell death [85]. Another piece of RNA sequencing data came from a recent study [86], where upregulated expression of MAPK-related genes was found in CD11b^mid^Ly6C^mid^Ly6G^lo^ M-MDSCs, generated in a co-culture system with mesenchymal stromal cells (MSCs) in the presence of GM-CSF, compared to monocytes defined as CD11b^hi^Ly6C^hi^Ly6G^lo^ [87]. Western blot data further revealed increased expression of phospho-JNK in these MDSCs, while p38 expression remained unchanged between the experimental and control groups. Pharmacological inhibition of JNK resulted in a failed induction of MDSC molecular features under MSC stimulation [86].

The critical role of p38 in MDSCs has been demonstrated recently by Alicea-Torres et al. [88]. They found that activated p38 signaling endowed neutrophils and monocytes with suppressive function by downregulating IFNAR1 in these cells. Previously, IFNAR1 has been shown as a key player in myeloid cells. As a component of IF1 signaling, IFNAR1 contributes to dendritic cell maturation and enhancement of antigen presentation, indicating the physiological function of this signaling axis. Interestingly, when exposed to tumor-derived factors and a hypoxic environment, p38 activation was more profound in PMN-MDSCs compared to M-MDSCs. Ubiquitin-mediated degradation of IFNAR1 was the molecular mechanism of the PMN-MDSCs’ suppressiveness. Taken together, different members of MAPKs may contribute to the suppressive phenotype of MDSCs depending on the stimulating factors and cells of origin.

## 3. Other Serine/Threonine Kinases

The STK family comprises approximately 300 members and represents the largest group among all protein kinases. Nearly every aspect of eukaryotic cellular function is regulated by the phosphorylation of proteins at serine and threonine residues. Dysregulation of STK signaling is frequently associated with a wide range of human diseases [89]. In addition to mTOR, MAPKs, and AMPK, as we discussed before, several other STKs have been reported to play critical roles in maintaining the immunosuppressive activity of MDSCs.

### 3.1. PERK

Numerous studies have demonstrated that MDSCs acquire an even stronger immunosuppressive phenotype in the TME compared to those in peripheral sites [90,91]. The TME exhibits distinct features compared to peripheral tissues, including hypoxia, nutrient deprivation, low pH, and oxidative stress due to the high level of free radicals. All of these factors activate endoplasmic reticulum (ER) stress responses in resident cells within the tumor mass. To survive such a hostile environment, MDSCs adapt their stress regulatory mechanisms [92]. Recently, Mohamed et al. [93] reported PKR-like endoplasmic reticulum (ER) kinase (PERK) as a molecular mechanism of such a process. They found that PERK was overexpressed in MDSCs derived from tumor tissues of mice and cancer patients compared to MDSCs derived from peripheral organs of a tumor host and immature myeloid cells from tumor-free mice [93]. As its name suggests, PERK is localized in the ER membrane and its primary function is to sense the unfolded proteins and trigger the unfolded protein response (UPR) by inhibiting general protein translation while selectively enhancing stress-related protein production [94]. PERK inhibition in MDSCs led to reduced immunosuppressive activity along with increased expressions of anti-tumor cytokines including IL-12 and TNF-α and enhanced CD8^+^ cell-mediated anti-tumor immunity [93]. The mechanism of action of this phenomenon is dependent on NRF2, a transcription factor that regulates cellular redox homeostasis [95]. Previously, constitutive activation of NRF2 has been associated with increased immunosuppressive function and survival of MDSCs by regulating oxidative stress in challenging environments [96,97].

### 3.2. PIM1

Adaptation to fatty acid metabolism is a crucial mechanism for the survival and proper function of various cell types, including myeloid cells and lymphocytes, particularly those residing in lipid-rich tissues and organs such as adipose tissue and the brain. For example, memory T cells, particularly tissue-resident memory T cells, rely on fatty acid uptake for long-term survival in lipid-rich environments, such as the skin [98]. Similarly, the TME is also enriched in lipids, suggesting that resident or recruited immune cells, such as MDSCs, must reprogram their metabolic profiles to adapt to these conditions. The kinase-dependent mechanism of lipid metabolism in MDSCs has been reported recently [99]. Xin et al. found that Proviral integration site for Moloney murine leukemia virus-1(PIM1)-expressing MDSCs in tumors are the possible reason for immune checkpoint blockade (ICB) resistance. Originally, PIM1 was established as a proto-oncogene and implicated in the initiation and progression of various cancer types [100]. Recent studies demonstrated that PIM1 also regulates immune cell functions [101]. Xin et al. found a strong correlation between PIM1 expression and fatty acid transport-related genes such as *Cd36* and *Gpr84* in myeloid cells from tumors through a single-cell RNA-sequencing analysis. Further in vitro and in vivo experiments showed that PIM1 is required for MDSCs to exert immunosuppressive activities. This and a subsequent report showed that the immunosuppressive phenotype of G-MDSCs but not M-MDSCs was dependent on PIM1 [99,102]. PIM1-mediated regulation of PPAR-γ through STAT3 phosphorylation at Ser727, but not the Tyr705 residue, was identified as the molecular mechanism of this suppressive activity [99]. PPAR-γ was recently found to be a critical component of the S100A4-mediated signaling axis that enhances macrophage polarization towards a pro-tumor (M2) phenotype [103]. Interestingly, the expression of PIM1 reached a significant level on day 7 (compared to 4-day MDSCs) in in vitro-generated MDSCs [99]. The authors explained that this phenomenon is due to the gradual enrichment of MDSCs. This may also imply that the complexity of the molecular mechanisms associated with immunosuppression increases with the chronicity of pathological conditions.

### 3.3. CaMKK2

The differentiation of myeloid progenitor cells into lineage-committed cells is essential for maintaining balanced physiological immune responses. Calcium/calmodulin-dependent serine/threonine kinase (CaMKK2) plays a critical role in the differentiation of hematopoietic progenitor cells [104]. For instance, downregulation of CaMKK2 is a key mechanism during normal granulopoiesis [105], and it controls the quiescence-associated signaling network in hematopoietic stem and progenitor cells (HSPCs) and its loss leads to improved hematopoietic regeneration following radiation injury [106], suggesting the possible involvement of CaMKK2 in the differentiation program of myeloid progenitor cells and that aberrant expression of this kinase may contribute to the dysregulated differentiation of myeloid progenitor cells. A recent study by Huang et al. [107] demonstrated that deletion of CaMKK2 in tumor-bearing mice inhibited MDSC expansion and accumulation while promoting the terminal differentiation of myeloid progenitors into more mature cell types such as dendritic cells and M1 macrophages. This shift enhanced T cell-mediated anti-tumor immunity and resulted in suppressed tumor growth [107]. CaMKK2 is overexpressed in multiple tumor types and associated with disease progression [108]. In brain tumor, CaMKK2 was upregulated in neurons and TAMs, and deficiency of CaMKK2 reprogrammed tumor-promoting TAMs to an immunostimulatory phenotype, improving the efficiency of immunotherapy [109]. Interestingly, deletion of CaMKK2 did not attenuate the immunosuppressive function of MDSCs but induced apoptosis [107], indicating that decreased tumor growth occurred not because of the diminished suppressive function of MDSCs but by impaired expansion of MDSCs, due to the loss of CaMKK2, in the tumor pathology. These data also imply that MDSC expansion and acquisition of suppressive functions are governed by distinct molecular mechanisms [110]. However, the role of CaMKK2 in macrophages is very delicate and plays both beneficial and deleterious roles depending on the context. Inhibition of CaMKK2 protects against harmful inflammation associated with obesity but may also impair the immune response to infections [111]. Therefore inhibitors or modulators of CaMKK2 should be designed to either enhance or suppress its activity, depending on the desired therapeutic outcome, such as reducing chronic inflammation or improving immune responses to infections.

## 4. Conclusions and Prospects

MDSCs are the major immunosuppressive populations within the tumor mass and peripheral tissues, undermining intrinsic anti-tumor responses and the efficacy of immunotherapeutic interventions. In this review, we discussed most, if not all, of the protein kinases that are directly required for the immunosuppressive functions of MDSCs in different cancer types and various pathological contexts (Table 1). Many of these kinases, recognized as tumor-promoting in cancer biology, are targets of existing or emerging therapies, highlighting their therapeutic potential [112,113,114,115]. However, the heterogeneity of MDSC populations and the influence of microenvironmental cues result in variable dependence on specific kinases for their immunosuppressive phenotype. These factors dynamically regulate kinase-mediated signaling pathways, shaping MDSC functionality.

Comparing protein kinase-mediated signal transduction pathways associated with the immunosuppressive phenotype in MDSCs derived from the same murine species but generated under different modeling systems, such as self-limited inflammatory conditions (e.g., acute liver injury) and MDSCs generated in sustained, chronic inflammatory contexts (e.g., tumor), may provide a clearer picture of the molecular mechanisms underlying the potent immunosuppressive functions of MDSCs in distinct pathophysiological contexts [67,80]. Such approach may also provide information on the basal level of the suppressive potential of myeloid cells that is required for regulating physiological inflammation [116,117]. Additionally, the intensity, duration, and degree of crosstalk with other signaling networks should be considered in such experimental designs. Another important aspect of the molecular mechanisms underlying the suppressive phenotype of MDSCs is the regulation of transcription factors. Protein kinases are important regulators of transcription factor function [118]. The different phosphoforms can be generated from the same transcription factors depending on the phosphorylated residue [119] or the different kinases may produce varying functional outcomes from the same transcription factor, as has been elucidated in the example of STAT3 [120,121,122].

Therefore, future research elucidating the interplay among kinase signaling networks, their transcriptional outputs and produced phosphoforms, and context-specific signaling dynamics will deepen our understanding of MDSC biology. Such insights are essential for developing targeted therapies that modulate MDSC function, ultimately enhancing anti-tumor immunity and improving immunotherapeutic outcomes.

## Figures and Tables

**Table 1:** Protein kinases associated with the immunosuppressive phenotype of MDSCs in different pathophysological conditions.

Protein Kinases	Models	Species	MDSC Phenotype	Transcription Factors	Roles	Pharmacological Inhibitors	Ref.
mTORC1	Murine tumor model, allogeneic transplant model	M	M-MDSC	NA	Rapamycin treatment or genetic deletion of mTORC1 decreased number of M-MDSCs and immunosuppressive function through inhibiting glycolysis.	Rapamycin	[33]
mTORC1	Murine tumor model	M	M-MDSC	NA	Rapamycin treatment decreased glycolysis and reduced the suppressive activities of M- MDSCs.	Rapamycin	[34]
mTORC1/mTORC2	Murine model of Pristane-induced lupus	M	M-MDSC	IRF-8	Metformin and mTOR inhibitors (rapamycin and NK128) decreased the percentage of M-MDSCs; adoptive transfer of metformin-treated M-MDSCs attenuated the disease symptoms.	Metformin, Rapamycin, NK128	[36]
mTORC1	Murine models of hepatic injury (CIH and PIH)	M	MDSC	HIF-1α	Rapamycin treatment potentiated the immunosuppressive function of MDSCs and mTORC1-deficient MDSCs exerted protective effects against immune-mediated hepatic injury and improved survival.	Rapamycin	[46]
mTORC1	Murine model of acute kidney injury	M	G-MDSC, M-MDSC	Rnux1	Rapamycin-treated MDSCs limited T-cell infiltration and significantly improved renal function; mTOR inhibition redirected MDSC differentiation towards the CD11b^+^Ly6G^+^Ly6C^low^ G-MDSC subset and enhanced their immunosuppressive activity.	Rapamycin	[47]
PI3Ks	HNSCC patient samples	H	M-MDSC	FOXO	PI3K inhibition reversed the immature phenotype to a more mature phenotype. T-cell suppression was rescued when co-cultured with PI3K-inhibited M-MDSCs.	Wortmanin, LY294002	[66]
PI3K-AkT	In vitro tumor infiltrating MDSC model	M	MDSC	NA	Akt inhibition led to the death of MDSCs.	X	[67]
PI3K δ/γ	Murine tumor models of head and neck cancers	M	G-MDSC	NA	IPI-145 treatment of G-MDSCs reduced suppressive ability and decreased Arg1 and Nos2 transcript levels but did not induce cell death.	IPI-145	[72]
MERTK	Murine model of lung transplantation	M	M-MDSC	NA	M-MDSCs with activated MERTK effectively facilitated the resolution of inflammation in ischemia/reperfusion injury by clearing the apoptotic neutrophils at injury sites, while M-MDSCs derived from MERTK^−/−^ mice failed to exhibit an immunosuppressive effect.	NA	[79]
TAM RTKs	Murine model of melanoma	M	MDSC	STAT3	The spleen MDSCs from Mertk^−/−^, Axl^−/−^, and Tyro3^−/−^ mice showed decreased expressions of suppressive mediators such as arginase-1, iNOS TGF-β, IDO, and ROS, and the functional assay was consistent with these results.	NA	[80]
ERK1/2 and JNK	LL2 tumor model	M	G-MDSC, M-MDSC	NA	Inhibition of ERK1/2 and JNK with SCH772984 and SP600125, respectively, increased apoptosis of both MDSC populations.	SCH772984 and SP600125	[84]
JNK	In vitro model	M	M-MDSC	NA	Pharmacological inhibition of JNK resulted in a failed induction of MDSC molecular features under MSC stimulation.	SP600125	[87]
p38	Murine tumor models, cancer patient samples	M, H	G-MDSC	IFNAR1	Activation of p38 was required for the suppressive functions of G-MDSC.	LY2228820	[88]
PERK	Murine tumor models, cancer patient materials	M, H	MDSC	NRF2	PERK inhibition in MDSCs reduced immunosuppressive activity along with increased expressions of anti-tumor cytokines.	GSK-2606414, AMG-44	[93]
PIM1	Bilateral tumor model	M	G-MDSCs	PPAR-γ, *p*-Ser727-STAT3	Required MDSCs’ immunosuppressive activities, caused ICB resistance.	AZD1208	[99]
CaMKK2	Lymphoma tumor model	M	MDSCs	NA	Deletion of Camkk2 induced terminal differentiation of MDSCs and reduced tumor growth.	STO-609	[107]

Notes: H, human; M, mouse; NA, not available.

## Abbreviations

AKI	Acute kidney injury
AMPK	AMP-activated protein kinase
BTK	Bruton’s tyrosine kinase
C/EBPβ	CCAAT/enhancer binding protein beta
CaMKK2	Calcium/calmodulin-dependent protein kinase kinase 2
CIH	Con A-induced immune-mediated hepatic injuries
DCs	Dendritic cells
DSKs	Dual-specificity kinases
ER	Endoplasmic reticulum
ERKs	Extracellular signal-regulated kinases
GAS6	Growth Arrest-Specific 6
GM-CSF	Granulocyte–macrophage colony-stimulating factor
HNSCC	head and neck squamous cell carcinoma
HSPCs	hematopoietic stem and progenitor cells
ICB	Immune checkpoint blockade
IDO	Indoleamine 2,3-dioxygenase
IF1	Inhibitory Factor 1
IFN-α	Interferon-alpha
IFNAR1	Interferon alpha and beta receptor subunit 1
IL-6	Interleukin-6
iNOS IRF-8	Inducible nitric oxide synthase Interferon regulatory factor-8
JNK	c-Jun N-terminal kinases
MAPKs	Mitogen-activated protein kinases
M-CSF	Macrophage colony-stimulating factor
mTOR	Mechanistic target of rapamycin
NETs	Neutrophil extracellular traps
NF-κB	Nuclear factor kappa B
2NBDG	2-(N-(7-Nitrobenz-2-oxa-1,3-diazol-4-yl)Amino)-2-Deoxyglucose
NK	Natural killer
NRTKs	Non-receptor tyrosine kinases
PERK	PKR-like endoplasmic reticulum (ER) kinase
PI3Ks	Phosphoinositide 3-kinases
PIH	Picryl chloride
PIM1	Proviral integration site for Moloney murine leukemia virus-1
PIP3	Phosphatidylinositol-3, 4, 5-triphosphate
PKB	Protein Kinase B
PROS1	Protein S
PPAR-γ	Peroxisome Proliferator-Activated Receptor gamma
PtdSer	Phosphatidylserine
ROS	Reactive oxygen species
RTKs	Receptor tyrosine kinases
STAT3	Signal transducer and activator of transcription 3
STKs	Serine/threonine kinases
TAM RTK	TAM (Tyro3, Axl, Mer) family of receptor tyrosine kinases
TAMs	Tumor-associated macrophages
TEK	Tyrosine kinase expressed in hepatocellular carcinoma
TGF-β	Transforming growth factor-beta
TLR7	Toll-like receptor 7
TKs	Tyrosine kinases
TME	Tumor microenvironment
UPR	Unfolded protein response
VEGF	Vascular endothelial growth factor
